# Ultrafast Volumetric Optoacoustic Imaging of Whole Isolated Beating Mouse Heart

**DOI:** 10.1038/s41598-018-32317-1

**Published:** 2018-09-20

**Authors:** Hsiao-Chun Amy Lin, Xosé Luís Déan-Ben, Michael Reiss, Verena Schöttle, Christian A. Wahl-Schott, Igor R. Efimov, Daniel Razansky

**Affiliations:** 1 Institute for Biological and Medical Imaging (IBMI), Helmholtz Center Munich, Neuherberg, Germany; 2 Faculty of Medicine, Technical University of Munich, Munich, Germany; 3 Center for Integrated Protein Science and Center for Drug Research, Department of Pharmacy, Ludwig Maximilians University of Munich, Munich, Germany; 4 Department of Biomedical Engineering, George Washington University, Washington, DC 20052 USA

## Abstract

The Langendorff-perfused heart technique has become the model of choice for multiparametric optical mapping of cardiac function and electrophysiology. However, photon scattering in tissues represents a significant drawback of the optical imaging approach, fundamentally limiting its mapping capacity to the heart surface. This work presents the first implementation of the optoacoustic approach for 4D imaging of the entire beating isolated mouse heart. The method combines optical excitation and acoustic detection to simultaneously render rich optical contrast and high spatio-temporal resolution at centimeter-scale depths. We demonstrate volumetric imaging of deeply located cardiac features, including the interventricular septum, *chordae tendineae*, and papillary muscles while further tracking the heart beat cycle and the motion of the pulmonary, mitral, and tricuspid valves in real time. The technique possesses a powerful combination between high imaging depth, fast volumetric imaging speed, functional and molecular imaging capacities not available with other imaging modalities currently used in cardiac research.

## Introduction

The technique of Langendorff-perfused heart isolation has led to major advancements in assessing cardiac mechanical and bioelectrical parameters by decoupling the influence of multiple factors complicating *in vivo* measurements, such as the autonomic nervous system and the hormone system^1,2^. The method involves quick cannulation of the excised heart and its retrograde perfusion via the aorta. In this way, cell viability and heart function are preserved by delivering nutrient-rich buffer solution to the entire heart through the coronary arteries, vascular bed, and veins. With careful maintenance of the pH and temperature of the perfusate, it is possible to maintain the isolated heart beating for several hours. With its relative simplicity as compared to *in vivo* setups, the Langendorff-perfused heart has become essential in many biomedical studies, providing access to physiologists, biochemists, and clinicians to better study heart biology. Moreover, continuous perfusion makes the method optimal for pharmacological testing of drug delivery further enabling studying the efficacy of genetic alterations and other therapeutic interventions combating ischemia injury^3–5^.

The isolated heart preparation ensures easy access by optical imaging that offers imaging speed, sensitivity, high spatial resolution, as well as a great variety of targeted moieties for the assessment of biophysical and biochemical parameters not available in the *in vivo* setting. For instance, voltage- and calcium- sensitive dyes have been used to map transmembrane potential and intracellular calcium transients^6–9^, popularizing optical imaging as a standard tool for the study of cardiac and epicardial electrophysiology. Advancements in optical imaging technology have enabled the acquisition of full planar views of the heart surface at a frame rate of several kilohertz^6,7^. In this way, rotor dynamics found in spiral propagation of potential across the heart has been studied to shed light onto atrial and ventricular arrhythmia and sudden cardiac death^10^. Most recently, the method has gained major significance due to the ability of simultaneous electro-mechanical optical and ultrasound mapping of membrane potential, intracellular calcium, and mechanical deformation, which provided the first experimental evidence for the existence of co-localized electrical and mechanical rotors during ventricular fibrillation^11^. However, while valuable information can be gathered from the epicardium, optical visualization of endocardium and other internal structures is prohibited due to intense light scattering in tissues. Chemical tissue clearing can facilitate high-resolution 3D observations through the entire heart with selective-plane microscopy techniques^12,13^, which is however not compatible with cell viability. Other modalities, such as magnetic resonance imaging (MRI) or computed tomography (CT), can alternatively be used to image the heart^14–16^. However, they mainly reveal anatomical tissue contrast while dynamic imaging in three dimensions further relies on gating approaches, impeding real-time imaging of perfusion and other rapid biological processes.

In this work, we devised a volumetric optoacoustic tomography (OAT) system to enable real-time functional imaging of the entire isolated Langendorff-perfused heart. The method synergistically combines optical excitation and acoustic detection to simultaneously render rich optical contrast and high resolution at centimeter-scale depths while further maintaining fast 3D imaging capacity crucial for beat-by-beat dynamic visualization of whole beating heart. The isolated heart allows for direct imaging of the heart without the presence of skin, ribs, and strongly absorbing blood, thus significantly improving the depth and image quality with respect to the *in vivo* imaging scenario^17,18^.

## Results

### Deep tissue imaging using OAT

**Figure 1:**
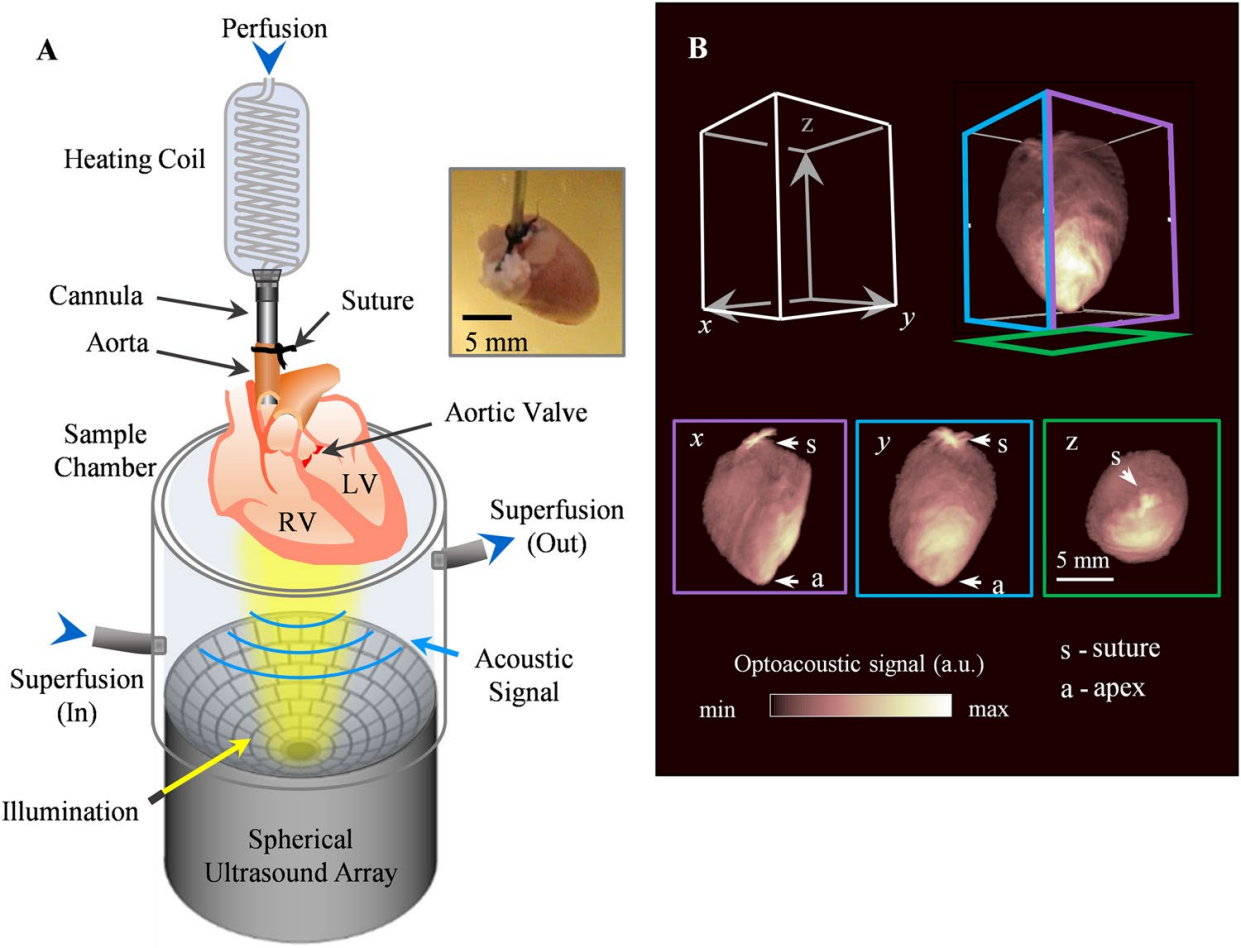
Imaging setup of real-time volumetric optoacoustic imaging of Langendorff-perfused heart. (**A**) The heart was attached to the output of the heating coil and perfused through the cannula. It was then suspended into the sample chamber from above. The perfusate filled the volume of the chamber, with input and output ports facilitating flow for sample superfusion. Short (10 ns duration) light pulses at 800 nm wavelength and 100 Hz pulse repetition frequency were delivered onto the heart from below while a spherical matrix ultrasound array was used to collect the generated optoacoustic responses. For each light pulse, a three-dimensional optoacoustic image was rendered, shown in (**B**), along with the maximum intensity projections (MIP) in *x*, *y*, and z dimensions. The Langendorff-perfused heart could be seen in its entirety, and the indicated features are the suture (s) and heart apex (a).

**Figure 2:**
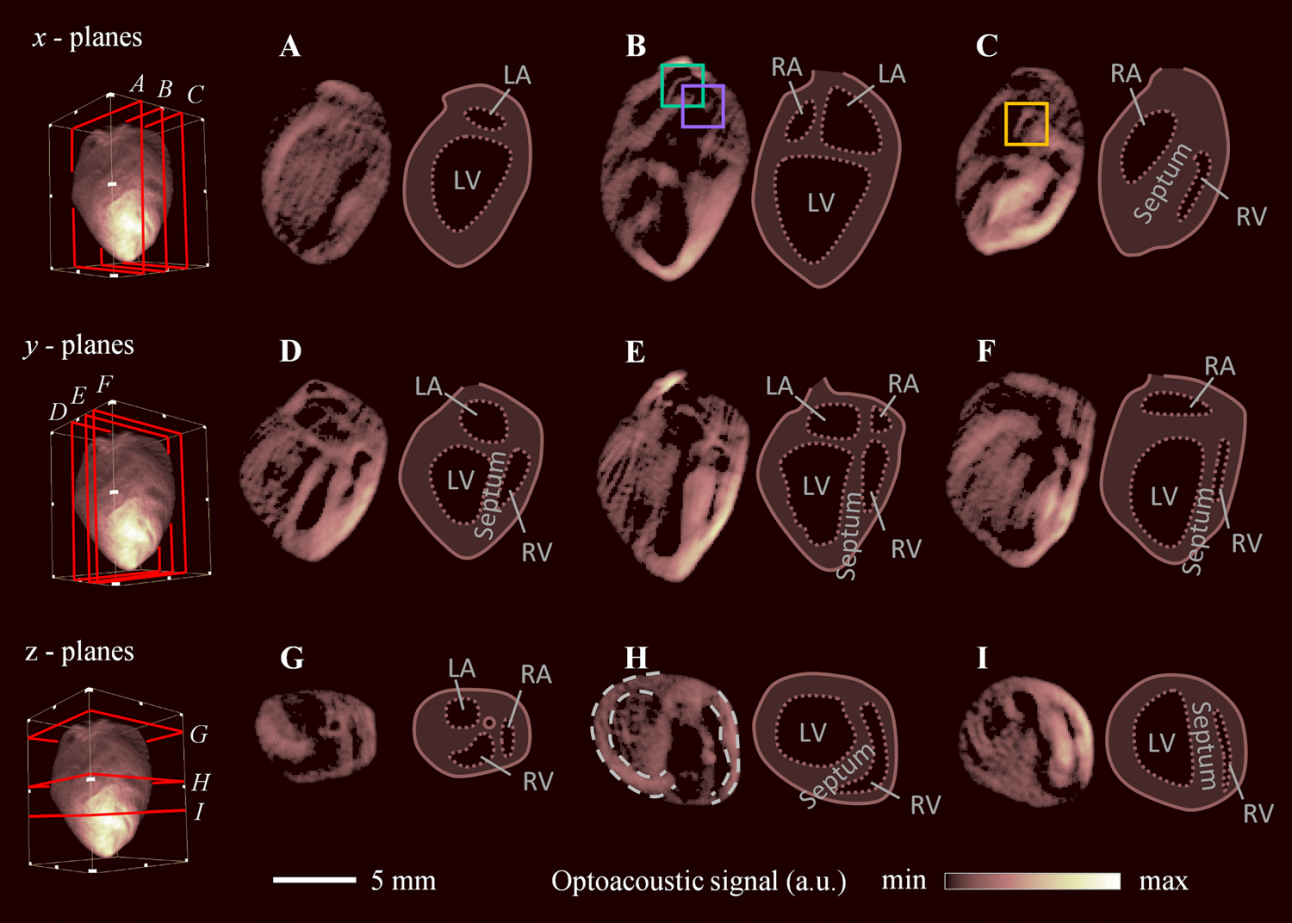
Cross-sectional images of the whole Langendorff-perfused heart. Single plane views were extracted along the *x* (**A**–**C**), *y* (**D**–**F**), and z (**G**–**I**) dimension. Features indicated in the side-by-side sketches include the left (LA) and right atria (RA), left (LV) and right ventricles (RV), and interventricular septum.

The volumetric OAT setup designed to optimally support imaging of entire isolated beating murine hearts is shown in Fig. 1A, consisting of a dedicated sample chamber, light illumination bundle and a spherical-matrix array for tomographic OA signal detection. The spherical array probe was attached to the sample chamber pointing upwards and tightly sealed at the edges (see Methods section for a detailed system description). After extraction, the hearts were cannulated and immediately moved into the sample chamber for imaging. A snapshot of the typical reconstructed volumetric image of the isolated heart is shown in Fig. 1B, where the *x*, *y*, and z views are displayed as maximum intensity projections (MIP). The outline of the heart along its long and short axis as well as the apex can be clearly identified. The black suture thread used during cannulation is a strong absorbing source that generates a bright spot in the images. The intensity distribution on the MIPs mainly corresponds to the light fluence distribution on the heart surface. The suspended heart was slightly tilted with the strongest OA signal on its lower right side corresponding to the excitation light spot. Yet, the excitation light was diffused across the heart volume and hence internal structures are clearly distinguished in the cross-sectional planes displayed in Fig. 2, where panel A–C, D–F, and G–I indicate the *x*, *y*, and z planes respectively. Most of the blood was washed out of the heart. Thus, OA contrast was provided by myoglobin and the small amount of hemoglobin remaining in the heart muscle capillaries.

Key structures can be resolved in the cross-sectional images, and the heart anatomy was characterized and marked in the side-by-side sketches, including the left (LA) and the right atria (RA), left (LV) and right ventricles (RV), and the interventricular septum. Other internal features that can be recognized include the papillary muscles and *trabeculae carneae*, which appear as ridge-like structures of the ventricular wall protruding into the lumen of the ventricle. Figure 2D–F closely resemble the four-chamber view well known from echocardiography, where the left and right ventricles appear on the left and right of the images respectively, separated by the septum. The surface of the ventricular septum can be seen in Fig. 2C. On the other hand, the atria appear before the ventricles scanning from top to bottom in the cross-sections along the z- direction (Fig. 2G–I). The image plane shown in Fig. 2H is normal to the heart surface and was used to characterize the heart wall thicknesses. The left and right ventricular walls were segmented (indicated by dotted lines), and their thickness medians and interquartile ranges were found to be 1.11 [1.02–1.20] mm and 0.63 [0.56–0.84] mm, respectively. Supplementary Movie S1 shows fly-throughs in the *x*, *y*, and z dimensions.

### Dynamic characterization of beating heart

**Figure 3:**
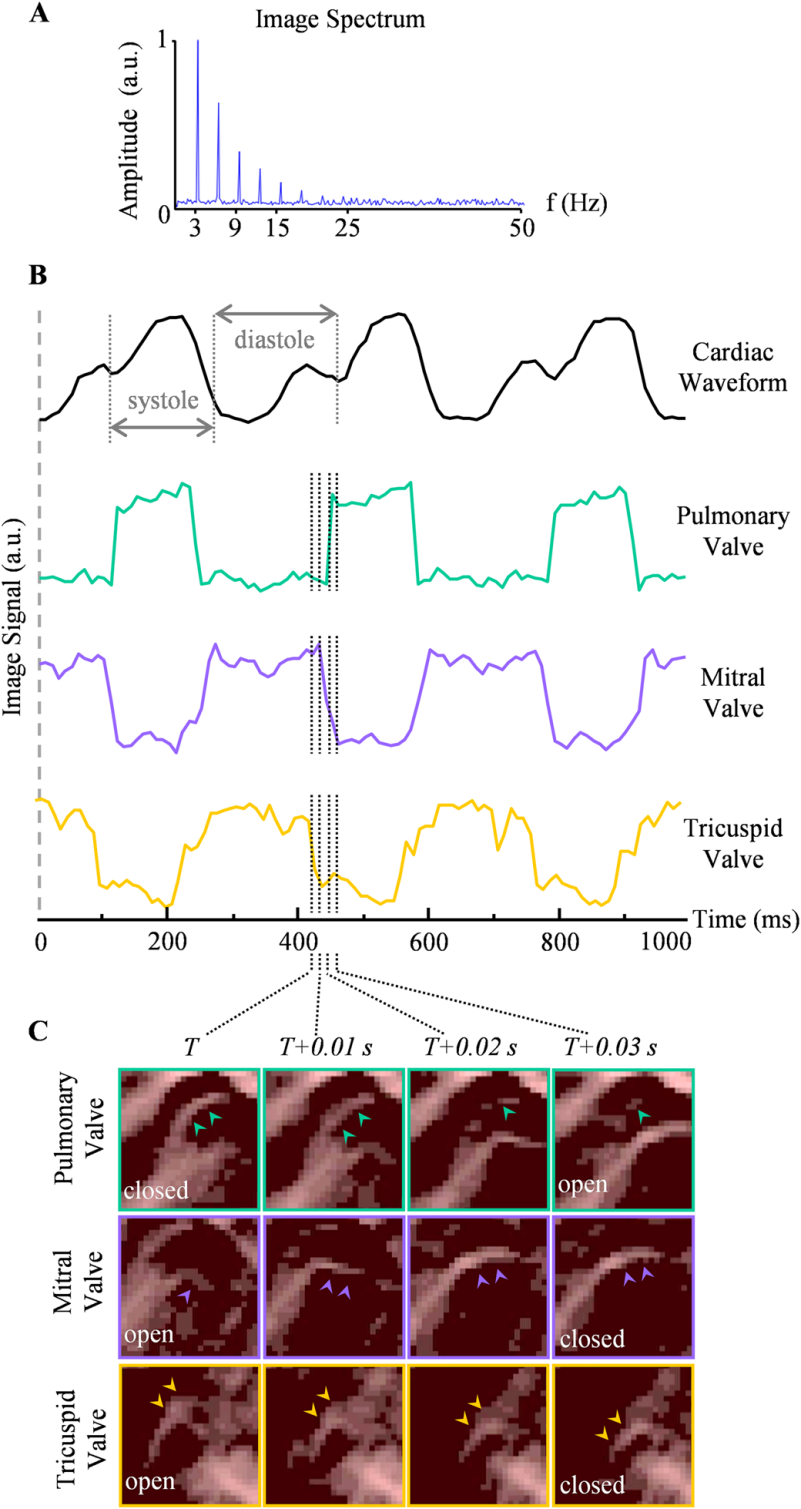
Heartbeat dynamics extracted from the time-lapse (4D) OAT data. (**A**) Image spectrum, obtained by summing the frequency spectra for all voxels within the FOV. The peak fundamental frequency at 3 Hz was assumed to be the heart rate. (**B**) Temporal profiles of OA signals from selected voxels sampled at 10 ms temporal resolution. The cardiac waveform was characterized by a voxel selected at the left ventricular wall, and the systole and diastole states are marked. Voxels selected at the pulmonary, mitral, and tricuspid valves are further shown. The semilunar valve was open during cardiac systole, in contrast to the two atrioventricular valves, which closed during systole and opened during diastole. The dotted lines indicate the transition between the open and close valves, consisting of four sampling events, and the cross-sectional close-up images (*y *- plane) of the respective valves corresponding to these time points are shown in (**C**). The corresponding locations of the image slices are indicated in Fig. 2B,C.

Information on the heart beat dynamics could be further extracted from the full 4D OA dataset by analyzing temporal profiles of the reconstructed images shown in Fig. 3. Figure 3A was obtained by summing the amplitudes of the Fourier transforms of the time-resolved OA signals in each individual voxel, thus representing an integrated spectral content within the entire imaged field of view (FOV). The image power spectrum consists of a peak frequency at 3 Hz, with the higher harmonics amplitudes tapering off in amplitude. As the dominant source of motion was attributed to the heart beat, the heart rate (~180 bpm) was estimated as the fundamental frequency in the Fourier spectrum. The beating Langendorff heart, presented as the MIPs, could be seen in Supplementary Movie S2. The cardiac events occurring during the heart cycle can be further observed in Fig. 3B, showing a temporal profile extracted from 1000 consecutive frames (10 ms time interval) for a voxel selected at the left ventricular wall.

With the retrograde perfusion, where fluid was constantly injected into the aorta, the aortic pressure was maintained, permanently closing the aortic valve. When scrutinizing the time-lapse 4D dataset, repeated motion due to opening and closing egged to the heart-beat cycles could lead to identification of the three other valves. Representative temporal profiles captured at various anatomical locations are displayed in Fig. 3C. The cardiac waveform was characterized by a voxel selected at the left ventricular wall, where the systole and diastole states are marked (Fig. 3B, top). Voxels at the pulmonary, mitral, and tricuspid valves were also analyzed, clearly revealing periodic motion. The reliable signal analysis was possible owing to the high penetration depth of the system. Specifically, the average signal-to-noise ratio (SNR) of the profiles shown in Fig. 3B was estimated to be ~10.8 (calculated by taking the optoacoustic signal of the valves relative to the background). The pulmonary valve opens at the beginning of systole and closes at the start of diastole. In contrary, the mitral valve and tricuspid valves were closed during systole, and open during diastole. In general, the valves oscillated between two binary states, with the atrioventricular valves being out of phase to the semilunar valve.

Furthermore, the high temporal resolution of the OA imaging system was sufficient to resolve the transient states, exemplified by the dotted lines in Fig. 3B. These four consecutive time points (*T* to *T* + 0.03 s) correspond to that of the temporal snapshots shown Fig. 3C, which are close-ups of the cross-sections (along the *y-* direction) of the pulmonary, mitral, and tricuspid valves, respectively. The corresponding positions in the whole-heart view are indicated in Fig. 2B,C. The leaflets of each valve are indicated by arrows in each time frame. The elongated structures of *chordae tendineae* and papillary muscles were also resolved in the mitral valve with Supplementary Movie S3 also showing its continuous motion.

## Discussion

This work presents the first implementation of an OA approach for 4D whole-organ imaging of a beating Langendorff-perfused heart. To this end, a wide range of mammalian species have been used in Langendorff-perfused heart preparations to study cardiac function in health and disease, including mice^8^, rats^19,20^, rabbits^9^, and larger animals^21^. Herein, the mouse model was adopted taking into consideration the abundance of the available disease models and the effective FOV of the OAT imaging system employed. The entire volume of the mouse heart could be captured in real time while the spatial resolution was adequate to resolve cardiac walls, valves, papillary muscles and even fine structured *chordae tendineae*.

Fundamentally, acoustic waves undergo several orders of magnitude less scattering as compared to light in biological tissues. Thus, the OA approach enabled herein resolving deep-tissue optical contrast with high spatial resolution, not attainable with fluorescence-based imaging methods. Volumetric OA tomography was recently employed for non-invasive imaging of the murine heart *in vivo*^17,18^. However, the effective penetration depth was limited to about 2–3 mm, mainly compromised by the vast amount of blood in the heart chambers causing strong light attenuation and amplitude decay of the OA signals versus depth. In addition, the lungs and the thorax are responsible for introducing severe distortions along the propagation path of the generated OA responses, subsequently leading to artifacts in the images obtained with standard acoustic inversion methods assuming a homogeneous acoustic medium^22,23^. Herein, it was shown that the basic imaging depth advantage of optoacoustics is further augmented by the lack of skin, ribs, lungs, and the whole blood, enabling the entire organ to be imaged accurately. Contrast was mainly provided by the residual hemoglobin in the myocardium, facilitating the visualization of deep tissue and exposing internal anatomy for investigation. For the first time to our knowledge, the interventricular septum could be observed with optical contrast in a Langendorff-perfused heart preparation.

The proof of concept experiments performed in this work relied on the excellent endogenous optical absorption contrast of the heart muscle, mainly provided by remaining hemoglobin molecules and other intrinsic tissue chromophores^24^. However, the approach is equally suitable for real-time visualization of targeted or genetically-encoded proteins, such as selectin-targeted probes^25^ or calcium-sensitive proteins that were recently successfully employed for OA imaging of neuronal activity^26^. Since calcium and voltage indicators similarly play a significant role in the understanding of myocardial physiology^27^, the newly introduced OA cardiac imaging approach may enable monitoring changes in these sensors in the entire heart. Calcium-dependent ionic currents are known to be implicated in the mechanisms of cardiac arrhythmias, cardiac myopathy, myocardial infarction and other conditions^28^. Four dimensional imaging of the entire heart with optical contrast can potentially provide a powerful tool to investigate the role of calcium in these conditions.

Another key characteristic of the presented method is its excellent temporal resolution. With the presently demonstrated 100 Hz volumetric acquisition rate determined by the 100 Hz PRF of the laser, real-time imaging on a beat-by-beat basis is possible without the need for cardiac gating. While such a high volumetric imaging speed is beyond reach for other bio-imaging modalities used in cardiac research, much faster 3D frame rates can be readily achieved as it only takes about 20 µs to record all the OA tomographic information from the entire mouse heart generated with a single laser shot. The powerful combination between high imaging depth, fast imaging speed, functional and molecular imaging capacities puts forth unprecedented new capabilities for cardiac research.

In conclusion, OA imaging of the entire Langendorff-perfused heart with high spatio-temporal resolution has been demonstrated. This represents an important milestone in the development of OA technology for cardiac imaging applications, which is poised to provide new insights into heart function in health and disease.

## Materials and Methods

All experiments were performed in full compliance with European laws on the protection of animals used for scientific purpose and the institutional guidelines of the Helmholtz Center Munich, and with approval from the Government District of Upper Bavaria.

### Langendorff heart preparation

We adopted a well-established Langendorff method of retrograde perfusion in murine hearts^8^. Tyrode’s solution^8^ (2 L) was pumped through via coronary artery as well as around the heart (superfusion). The solution was oxygenated by carbogen gas (95%/5% O_2_ to CO_2_ ratio) and its pH was maintained at 7.35 ± 0.05. Separate water baths (153–1071, Thermo Scientific GmbH, Munich, Germany) were used to maintain the Tyrode’s solution at 37 °C for the perfusion and superfusion circuits. CD1 mice were overdosed with ketamine (150 mg/kg) and xylazine (10 mg/kg) to harvest their hearts. Once the animal exhibits no pain reflex, established by pinching the paw, the thoracic cavity was opened to excise the heart. A 21-gauge cannula filled with perfusion buffer was inserted in the ascending aorta and fixed using 4-0 black-braided silk sutures (Resorba GmbH, Nuremberg, Germany).

### Optoacoustic tomography (OAT) set-up

The cannulated heart was immediately moved into the imaging system containing sample chamber, illumination fiber bundle and a spherical-matrix detection array. The spherical array probe was attached to the sample chamber pointing upwards and tightly sealed at the edges. The volume enclosed in the chamber was superfused with the Tyrode’s solution, which further facilitated acoustic coupling to the detection array. The heart was attached to the output of a heating coil providing the perfusion fluid and suspended into the sample chamber from above. An automatic pump (PERIPRO-4LS, World Precision Instruments, Sarasota, USA) controlled the perfusion flow rate at 5 mL/min. Superfusion of the heart was maintained at a constant flow of 70 mL/min by a second pump (15104696, Thermo Fisher Scientific GmbH, Munich, Germany). The perfusion was initiated at least 10 minutes before the measurement to wash out remaining blood and allow the heart to stabilize.

### Optoacoustic (OA) imaging set-up

Volumetric imaging of the beating heart was performed by illuminating it with ~10 ns duration pulses of light at 800 nm wavelength generated by an optical parametric oscillator (OPO) laser (Innolas Laser GmbH, Krailling, Germany) running at pulse repetition frequency (PRF) of 100 Hz. A custom-made fiber bundle (CeramOptec GmbH, Bonn, Germany) was used to guide the laser output through the center of the spherical array probe, thus illuminating the sample from the bottom with a fluence of ~20 mJ/cm^2^. The broadband optoacoustic (OA) responses generated in the imaged sample were captured by the 512 individual piezocomposite elements (5 MHz central frequency, ~100% detection bandwidth) distributed on a concave spherical surface of the custom-made matrix array covering an angle of 140° (1.3 π solid angle)^18^. The system provides an approximately isotropic resolution of 150 μm around the center of the sphere. Following each laser pulse, the OA signals captured by all the channels were simultaneously sampled by a custom-made multi-channel digitizer (Falkenstein Mikrosysteme GmbH, Taufkirchen, Germany). 1000 consecutive volumetric image frames were recorded at a rate of 100 Hz, corresponding to a total acquisition duration of 10 s.

### Image reconstruction

The acquired image sequence was processed off-line. No signal averaging was performed. Prior to image reconstruction, the acquired temporal signals were first deconvolved by the electrical impulse response of the detection array^29^. Subsequently, band-pass filtering with a second-order Butterworth filter having cut-off frequencies of 0.1 and 7 MHz was performed to remove low frequency bias and high frequency noise outside the effective transducer bandwidth. 3D OA images on a grid of 150 × 150 × 150 voxels (15 × 15 × 15 mm^3^), covering the entire mouse heart, were rendered for each laser pulse using a GPU-accelerated back projection algorithm^30^. The reconstruction time for each 3D frame was approximately 10 ms.

## Electronic supplementary material

Supplementary Movie 1

Supplementary Movie 2

Supplementary Movie 3

Supplemental Materials

## Data Availability

The datasets generated during and analyzed during the current study are available from the corresponding author on reasonable request.

## References

[1] Skrzypiec-Spring M (2007). Isolated heart perfusion according to Langendorff—still viable in the new millennium. J Pharmacol Toxicol Methods..

[2] Bell RM (2011). Retrograde heart perfusion: the Langendorff technique of isolated heart perfusion. J Mol Cell Cardiol..

[3] Rossello X (2016). Characterization of the Langendorff perfused isolated mouse heart model of global ischemia–reperfusion injury: impact of ischemia and reperfusion length on infarct size and LDH release. J Cardiovasc Pharmacol Ther..

[4] Ong SB (2017). Nanoparticle delivery of mitoprotective agents to target ischemic heart disease. Future Cardiol..

[5] Olenchock BA (2016). EGLN1 inhibition and rerouting of α-ketoglutarate suffice for remote ischemic protection. Cell..

[6] Efimov IR (2004). Optical imaging of the heart. Circ Res..

[7] Nanthakumar K (2007). Optical mapping of Langendorff-perfused human hearts: establishing a model for the study of ventricular fibrillation in humans. Am J Physiol Heart Circ Physiol..

[8] Lang D (2011). Optical mapping of action potentials and calcium transients in the mouse heart. J Vis Exp..

[9] Lou Q (2011). Multiparametric optical mapping of the Langendorff-perfused rabbit heart. J Vis Exp..

[10] Pandit SV, Jalife J (2013). Rotors and the dynamics of cardiac fibrillation. Circ Res..

[11] Christoph J (2018). Electromechanical vortex filaments during cardiac fibrillation. Nature..

[12] Keller PJ, Dodt HU (2012). Light sheet microscopy of living or cleared specimens. Curr Opin Neurobiol..

[13] Ding Y (2017). Light-sheet fluorescence imaging to localize cardiac lineage and protein distribution. Sci Rep..

[14] Zheng Y. *et al*. Four-chamber heart modeling and automatic segmentation for 3D cardiac CT volumes. *Proc. SPIE 6914, Medical Imaging 2008: Image Processing*. **691416** (2008). 10.1109/TMI.2008.2004421 18955181

[15] Ruff J (2000). Magnetic resonance imaging of coronary arteries and heart valves in a living mouse: techniques and preliminary results. J. Magn. Reson..

[16] Trayanova NA (2011). Whole heart modeling: Applications to cardiac electrophysiology and electromechanics. Circ. Res..

[17] Deán-Ben XL (2015). High-frame rate four dimensional optoacoustic tomography enables visualization of cardiovascular dynamics and mouse heart perfusion. Sci Rep..

[18] Lin HC (2017). Characterization of cardiac dynamics in acute myocardial infarction model by four-dimensional optoacoustic and magnetic resonance imaging. Theranostics..

[19] Hearse DJ, Sutherland FJ (2000). Experimental models for the study of cardiovascular function and disease. Pharmacol Res..

[20] Sutherland FJ, Hearse DJ (2000). The isolated blood and perfusion fluid perfused heart. Pharmacol Res..

[21] Schechter M.A. *et al*. An isolated working heart system for large animal models. *J Vis Exp*. **88** (2014). 10.3791/51671 PMC4189428 24962492

[22] Poudel J (2017). Mitigation of artifacts due to isolated acoustic heterogeneities in photoacoustic computed tomography using a variable data truncation-based reconstruction method. J Biomed Opt..

[23] Dean-Ben XL (2011). Statistical approach for optoacoustic image reconstruction in the presence of strong acoustic heterogeneities. IEEE Trans Med Imag..

[24] Weber J (2016). Contrast agents for molecular photoacoustic imaging. Nat Methods..

[25] Taruttis A (2013). Multispectral optoacoustic tomography of myocardial infarction. Photoacoustics..

[26] Deán-Ben XL (2016). Functional optoacoustic neuro-tomography for scalable whole-brain monitoring of calcium indicators. Light Sci Appl..

[27] Swynghedauw B (1999). Molecular mechanisms of myocardial remodeling. Physiol Rev..

[28] Salama G, Hwang SM (2009). Simultaneous optical mapping of intracellular free calcium and action potentials from Langendorff perfused hearts. Curr Protoc Cytom..

[29] Rosenthal A (2011). Optoacoustic methods for frequency calibration of ultrasonic sensors. IEEE Trans Ultrason Ferroelect Freq Control..

[30] Deán-Ben XL (2013). Volumetric real-time tracking of peripheral human vasculature with GPU-accelerated three-dimensional optoacoustic tomography. IEEE Trans Med Imag..

