# Supplementary material for: Ultrafast Volumetric Optoacoustic Imaging of Whole Isolated Beating Mouse Heart
Source: Sci Rep. 2018 Sep 20;8:14132. doi: 10.1038/s41598-018-32317-1 (PMC6148063; doi:10.1038/s41598-018-32317-1)
Supplement: Supplementary file 4 — Supplemental Materials [file 41598_2018_32317_MOESM4_ESM.pdf]

Ultrafast Volumetric Optoacoustic Imaging of Whole Isolated Beating Mouse Heart

Hsiao-Chun Amy Lin[1,2], Xosé Luís Déan-Ben[1], Michael Reiss[1,2], Verena Schöttle[3], Christian A. Wahl-Schott[3], Igor R. Efimov[4], and Daniel Razansky[1,2]*

1. Institute for Biological and Medical Imaging (IBMI), Helmholtz Center Munich, Neuherberg, Germany
2. Faculty of Medicine, Technical University of Munich, Germany
3. Department of Pharmacy, Center for Drug Research and Center for Integrated Protein Science Munich, Ludwig-Maximilians-Universität München, Munich, Germany
4. Department of Biomedical Engineering, George Washington University, Washington, DC 20052 USA.

* Corresponding author:

Daniel Razansky

Address	: Ingolstaedter Landstrasse 1, 85764, Neuherberg, Germany
Telephone	: +49 89 3187 1587
Fax	: +49 89 3187 3017
Email	: dr@tum.de

SUPPLEMENTAL MATERIALS

VIDEO LEGENDS

Supplementary Movie 1

The Langendorff heart at a single timepoint, displayed as cross-sectional fly-throughs. Projections in the x, y, and z dimensions are shown sequentially, with 100 µm steps. Scalebar: 2 mm.

Supplementary Movie 2

The beating Langendorff heart shown as the maximum intensity projection (MIP), acquired with 100 ms temporal resolution. Scalebar: 5 mm.

Supplementary Movie 3

Temporal movie of x, y, and z cross-sections selected planes demonstrating mitral valve dynamics. The movie shows valve opening- and closing- events, with 100 ms temporal resolution. Scalebar: 5 mm.
